# Six-minute walk test reveals delayed oxygen uptake kinetics in ischemic cardiomyopathy

**DOI:** 10.1590/1414-431X2024e14276

**Published:** 2025-02-03

**Authors:** I.S. Rocco, B.C. Matos-Garcia, M. Viceconte, C. Bublitz, F.S. Menezes-Rodrigues, F.S. Tallo, R.M. Arida, W.J. Gomes, N.A. Hossne, R. Arena, S. Guizilini

**Affiliations:** 1 Programa de Pós-Graduação em Cardiologia, Escola Paulista de Medicina, Universidade Federal de São Paulo, São Paulo, SP, Brasil; 2 Disciplina de Cirurgia Cardiovascular, Escola Paulista de Medicina, Universidade Federal de São Paulo, São Paulo, SP, Brasil; 3 Department of Physical Therapy, College of Applied Health Sciences, University of Illinois, Chicago, IL, USA; 4 Programa de Pós-Graduação em Ciência Cirúrgica Interdisciplinar, Escola Paulista de Medicina, Universidade Federal de São Paulo, SP, Brasil; 5 Disciplina de Clinica Médica, Escola Paulista de Medicina, Universidade Federal de São Paulo, São Paulo, SP, Brasil; 6 Departamento de Fisiologia, Universidade Federal de São Paulo, São Paulo, SP, Brasil

**Keywords:** Cardiac rehabilitation, Cardiometabolic risk factors, Coronary disease, Coronary artery disease, Heart failure, Systolic

## Abstract

Although correlated to peak oxygen uptake (VO_2_), the six-minute walk test (6MWT) alone cannot provide precise physiological insight regarding the specific cause(s) of exercise limitations. We aimed to analyze whether 6MWT is able to properly detect differences in the cardiorespiratory responses between patients with stable coronary artery disease (SCAD) and those with ischemic cardiomyopathy (IC) and determine whether the degree of abnormality in ejection fraction is related with impaired submaximal exercise capacity. Twenty-two subjects with SCAD and 19 subjects with IC underwent a 6MWT while simultaneously using a mobile telemetric cardiopulmonary monitor to assess cardiorespiratory responses. VO_2_ response at exercise onset was used to obtain VO_2_ on-kinetics, and the slope of ventilation *vs* carbon dioxide output (VE/VCO_2_) was calculated. IC subjects exhibited significantly delayed VO_2_ on-kinetics compared with the SCAD group (P<0.01) and higher VE/VCO_2_ slope (IC=40.45 [95%CI: 39.76 to 41.1] *vs* SCAD=34.36 [95%CI: 34.03 to 34.69], P=0.001). The left ventricular ejection fraction (LVEF) was moderately correlated with VO_2_ on-kinetics in the SCAD group, but no relationship was found in the IC group. Pulmonary function was correlated with the VE/VCO_2_ slope only in the IC group. Subjects with IC presented slower VO_2_ on-kinetics during the 6MWT than those with SCAD. Once reduction in left ventricular function is achieved, LVEF had no association with exercise capacity. Pulmonary function could help identify IC patients at risk of ventilatory inefficiency and may add diagnostic power to the 6MWT.

## Introduction

Coronary artery disease (CAD) leads to a mismatch between myocardial oxygen supply and demand, resulting in ischemic cardiomyopathy (IC) and reduced left ventricular ejection fraction (1,2). Exercise capacity is classically evaluated through the gold standard of cardiopulmonary exercise testing (CPET), which can measure oxygen uptake (VO_2_) at maximum effort (3). However, most activities of daily living are performed at a submaximal level of exertion. Therefore, the 6-minute walk test (6MWT) has been described as more representative of functional capacity because it involves a moderate level of exertion that approximates the lactate threshold (4). Although the 6MWT is correlated to peak VO_2_ (5), it alone cannot provide precise physiological insight regarding the specific cause(s) of exercise limitations. The simultaneous use of mobile telemetric cardiopulmonary monitoring (MOB) while performing the 6MWT could add diagnostic power of the classic CPET by obtaining breath-by-breath ventilatory expired gas responses.

The constant walking speed reached during the 6MWT also allows the assessment of the VO_2_ transition from rest to exercise, defined as VO_2_ on-kinetics (6). The VO_2_ on-kinetics has been found to be delayed in heart failure (HF) with reduced ejection fraction, and more recently also in HF with preserved ejection fraction (7,8). Beyond peak VO_2_, studies show that the VO_2_ on-kinetics appear to be a better prognostic predictor for HF patients (9), as is the ventilatory efficiency. Hoshimoto-Iwamoto et al. (10) standardized the evaluation of ventilatory efficiency slope through a submaximal test.

Ventilatory efficiency slope is related to impairment in ventilation volume caused by pulmonary congestion secondary to left ventricular failure. Patients with IC could have an alteration of the pulmonary function related to low stroke volume. Thus, we hypothesized that individuals with IC would present delayed VO_2_ on-kinetics and worse ventilatory parameters during the 6MWT compared to stable CAD (SCAD) patients without significant ventricular alterations.

To address these issues, the aims of the current study were to: 1) analyze whether 6MWT is able to properly detect differences in the cardiorespiratory responses between SCAD and IC subjects; and 2) determine whether the degree of abnormality in ejection fraction is related with impaired submaximal exercise capacity.

## Material and Methods

### Subject characteristics

**Figure 1:**
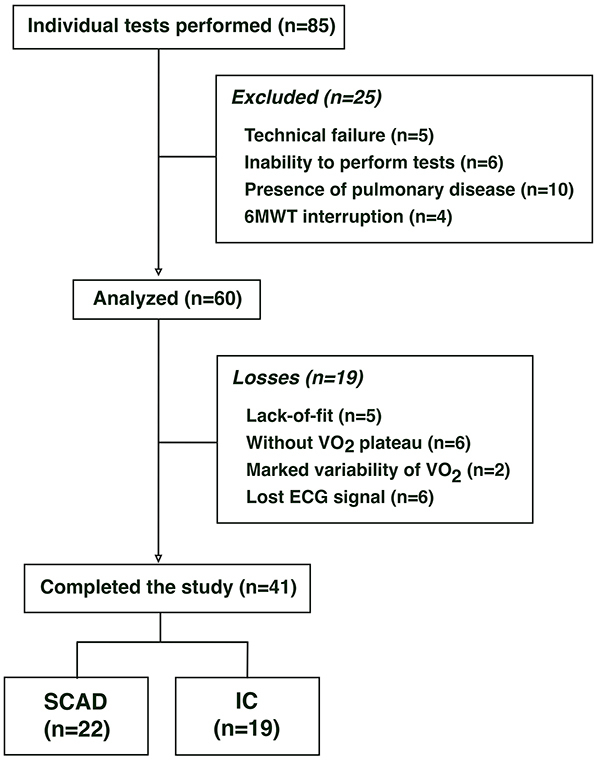
Flow-chart of study analyses. 6MWT: six-minute walk test; ECG: echocardiogram; SCAD: stable coronary artery disease; IC: ischemic cardiomyopathy.

Forty-one volunteers were enrolled in this study: 19 subjects with IC and reduced or moderately reduced ejection fraction, i.e. LVEF <45% (1), and 22 subjects with SCAD with preserved static ventricular function, i.e. those who did not develop diastolic or systolic dysfunction and no signs of HF syndrome (Figure 1). The study was approved by the institution’s Ethics Committee and all subjects signed an informed consent (number 1.424.088).

CAD diagnosis was confirmed by coronary angiography, in which all subjects had multivessel coronary artery involvement under optimal medical therapy. The left ventricular ejection fraction (LVEF) was determined using echocardiography (1). Sedentary behavior was defined according to the WHO guidelines on physical activity and sedentary behavior (11). Exclusion criteria included recent myocardial infarction (<6 months), angina or chest discomfort, presence of chronic or acute pulmonary disease, chronic kidney disease, neurological or orthopedic restrictions, morbid obesity (body mass index ≥40 kg/m^2^), hemodynamic instability or severe arrhythmias during the test, inability to understand and/or perform the tests (defined as patients unable to follow instructions or with gait restrictions, such as the use of gait assistive device), and evidence of lack-of-fit in the VO_2_ analysis. Patients were recruited from a myocardial outpatient care clinic, in which patients with coronary artery disease are treated at different stages.

### Submaximal exercise capacity protocol

The 6MWT was performed to assess the submaximal exercise capacity, according to the American Thoracic Society guidelines (4). The prediction equation proposed by Soares et al. (12) was used to predict walking distances for all subjects. The 6MWT was performed on a flat 30 m corridor and the distance achieved during the 6-minute test was recorded in meters. Two tests were performed to assess the learning effect of the test and familiarize patients with the equipment. The perceived exertion Borg scale (PEB) for dyspnea and leg fatigue was applied before, after, and every 2 min during the 6MWT. The ATS criteria were used for test interruption (4): intolerable dyspnea (PEB 7), angina or chest pain, pallor, dizziness, vertigo, palpitation, leg cramps or severe lower limb fatigue (PEB>7), oxygen desaturation (SpO_2_<89%), abnormal gait pattern or uncoordinated gait, and loss of balance.

### Measurement of cardiopulmonary response and gas exchange

Mobile telemetric cardiopulmonary monitoring (Oxycon Mobile-Viasys Healthcare, USA) was used during the 6MWT to measure real time breath-by-breath cardiorespiratory responses (6). Subjects carried the 950-g equipment with data storage and transfer units on a harness; this additional weight had no effect on walking distance (6). Baseline VO_2_ was measured until stable values were achieved during rest for at least 3 min by averaging the last minute prior to exercise. Heart rate (HR) was recorded beat-by-beat with a 12-lead electrocardiogram, the arterial O_2_ saturation was estimated from the earlobe by pulse oximetry (Oxycon Mobile-ECG module/Oxymeter, Viasys Healthcare, USA), and blood pressure was measured using a sphygmomanometer. Tidal volumes and breathing frequency were continuously sampled by a facemask (with a dead space <70 mL) linked to a turbine volume transducer. Breath-by-breath calculations of VO_2_ and carbon dioxide output (VCO_2_) were digitized. Steady-state variables were obtained as the mean of the final 3 min of the test. An established index was calculated as the slope of the relationship between minute ventilation (VE) and VCO_2_ (VE/VCO_2_ slope) (10). The VE/VCO_2_ slope of a constant load test is similar to the CPET when calculated from the start to the 4th minute, when these variables present a similar fit.

Original breath-by-breath data were imported and averaged over consecutive periods of 15 s. A monoexponential regression model was used to fit the exercise onset curve according to the following equation (13): f(t) = y0 + (y1-y0) (1-e^-t/*τ*^) with the ‘f(t)' indicating VO_2_ at a certain time (t); ‘y0' as the lower limit at t=0, indicating the VO_2_ resting value; ‘y1' as the upper limit, i.e. VO_2_ steady-state (VO_2SS_); and ‘*τ*' as the time constant, i.e. the time taken to reach 63% of the function.

The short time delay commonly indicated as phase I was not modeled in this study, as a previous report demonstrated no distinction from the second exponential phase (14). Moreover, the time delay entails a potential risk of overestimation of the *τ*. As time delay was not calculated, *τ* equals the mean response time (MRT), representing the time needed to achieve 63% of VO_2SS_.

As described in previous studies (6,15), a re-parametrization of MRT was calculated by correcting MRT for work rate (wMRT) during the 6MWT to avoid possible differences in intensity. The work rate was obtained using the difference between VO_2_ at rest and during effort, as follows: wMRT = MRT × (VO_2SS_ − VO_2rest_).

The goodness of fit was first visually assessed by three independent investigators in order to detect obvious fitting deficiencies, 5 of which were excluded (Figure 1). The goodness of fit for the nonlinear regression was tested for each of the remaining curves. Five curves had a poor fit and were excluded (i.e. where the R squared remained below 0.80).

### Pulmonary function

Forced vital capacity (FVC) was obtained using a portable spirometer (Spirobank G, MIR, Italy) according to the standards of the American Thoracic Society (16). Also, the forced expired volume in 1 s was evaluated to exclude patients with acute or chronic obstructive lung disorders.

### Statistical analysis

Categorical data are reported in absolute (n) and relative (%) frequency. Semi-continuous and continuous variables are reported as means±SD. Variables were tested for normal distribution by applying the Shapiro-Wilk test. Differences between subjects were assessed by the general linear model, Mann-Whitney U, or chi-squared test as appropriate. The Pearson correlation test was used to investigate the association between VO_2_ on-kinetics with LVEF. A P-value <0.05 was considered statistically significant for all tests. A *post hoc* sample size calculation was performed to obtain the power of analysis with GPower software. Statistical analyses were performed using Statistica Software.

## Results

### Sample characteristics

A flow-chart of the study is shown in Figure 1. The clinical and anthropometric characteristics of the participants are described in Table 1. The study groups were homogeneous with respect to anthropometrics and baseline clinical data, except for body weight, LVEF, and 6MWT distance (Table 1).

**Table 1:** Anthropometrics and functional capacity according to groups.

	SCAD(n=22)	IC(n=19)	P value
Age (years)^t^	59.64±1.39	61.00±1.63	0.52
Male/Female (n)^f^	19/3	14/5	0.31
Height^t^	1.63±0.08	1.61±0.08	0.63
Weight^t^	70.2±9.8	64.1±6.1	0.02
BMI (kg/m^2^)^t^	27.45±0.79	25.87±1.28	0.28
Hypertension, n (%)^f^	18 (81.8)	16 (84.2)	0.83
Diabetes, n (%)^f^	5 (22.7)	8 (42.1)	0.18
Peripheral artery disease, n (%)^f^	1 (4.5)	1 (5.2)	1.00
Current smokers^f^	3 (13.6)	2 (10.5)	0.76
Smoking load (pack-years)	32.3±10.7	35±3.5	0.78
Sedentary behavior^f^	17 (77.3)	16 (84.2)	0.70
Previous MI, n (%)^f^	11 (50.0)	15 (78.9)	0.05
Main affected artery			
LAD, n (%)^f^	9 (40.9)	9 (47.4)	0.67
RCA, n (%)^f^	9 (40.9)	7 (37.2)	0.79
Others, n (%)^f^	3 (13.6)	2 (10.5)	0.76
Stenosis degree (%)^u^	74.8±4.5	77.1±3.3	0.69
LVEF (%)^t^	0.62±0.23	0.37±0.21	<0.001
β-blockers (mg/day)^t^	81.9±9.5	84.2±7.9	0.85
6MWT (m)^t^	468.8±14.9	405.1±15.3	0.016
% Predicted^t^	82±10	70±7	0.01
D*W (km·kg)^t^	32.9±8.3	25.9±5.7	0.004

Data are reported as means±SD, unless otherwise noted. ^t^Student's *t*-test; ^f^Fisher's exact test; ^u^Mann-Whitney U test. β-block: β blockers medication doses; *τ*: time constant; 6MWT: 6-minute walk test; BMI: body mass index; D*W: distance and body-weight product; LAD: left anterior descendent; LVEF: left ventricular ejection fraction; MI: myocardial infarction; RCA: right coronary artery.

### Cardiorespiratory responses in the 6MWT: SCAD *vs* IC

All individuals had significantly increased HR, VO_2_, VCO_2_, and VE during the steady state of the 6MWT compared with baseline values, i.e. at rest. When the groups were compared at steady state, those with IC reached a higher VE/VCO_2_ and lower VCO_2_ than the SCAD group (P=0.042 and P=0.002, respectively). Moreover, significantly lower values of VO_2_ were achieved during the 6MWT by subjects with IC compared with the SCAD group (P=0.004). Subjects with IC presented lower VO_2_/HR compared with the SCAD group (P=0.017, Table 2).

**Table 2:** Gas-exchange and cardiopulmonary responses during 6-minute walk test according to groups.

	SCAD (n=22)		IC (n=19)	
	Rest	Steady-state	Rest	Steady-state
HR (bpm)	61.73±9.10	89.49±27.80*	67.23±11.80	91.33±20.77*
VO_2_/HR (mL·bpm^−1^)	4.5±1.3	10.8±3.1*	3.7± 1.1	7.9±2.7*†
VO_2_ (mL·min^−1^)	280±57	962±276*	249±41	728±145*†
VO_2_ (mL·min^−1^·kg^−1^)	3.98±0.79	13.73±3.39*	3.89±0.62	11.36±2.15*†
VCO_2_ (mL·min^−1^)	244±43	940±272*	224±53	682±155*†
RER	0.88±0.10	0.97±0.10*	0.89±0.11	0.94±0.07*
METS	1.1±0.2	4.0±0.8*	1.1±0.2	3.3±0.6*†
VE (L·min^−1^)	9.95±2.34	34.28±10.87*	9.70±2.73	27.70±6.71*
VE/VO_2_	35.61±5.86	36.33±6.72	38.79±8.18	38.06±6.15
VE/VCO_2_	40.36±4.65	36.57±4.41*	43.10±6.33	40.72±5.29*†
BR (%)	86.80±5.40	57.93±20.51*	83.01±8.05	55.08±21.59*
PEB (dyspnea)	0.10±0.31	2.65±2.35*	0.14±0.24	3.17±2.40*
PEB (limb fatigue)	0.05±0.23	2.15±1.82*	0.12±0.48	2.69±2.31*

Data are reported as means±SD. General linear model for repeated measures with Bonferroni *post hoc* test was applied in this analysis. *P<0.01 steady-state *vs* rest; †P<0.05 IC *vs* SCAD. BR: breathing reserve; HR: heart rate; METS: metabolic equivalents; PEB: perceived exertion Borg Scale; RER: respiratory exchange ratio; VCO_2_: carbon dioxide output; VE: ventilation; VE/VO_2_: ventilatory equivalent of oxygen; VE/VCO_2_: ventilatory equivalent of carbon dioxide; VO_2_: oxygen uptake; VO_2_/HR: oxygen pulse.

**Figure 2:**
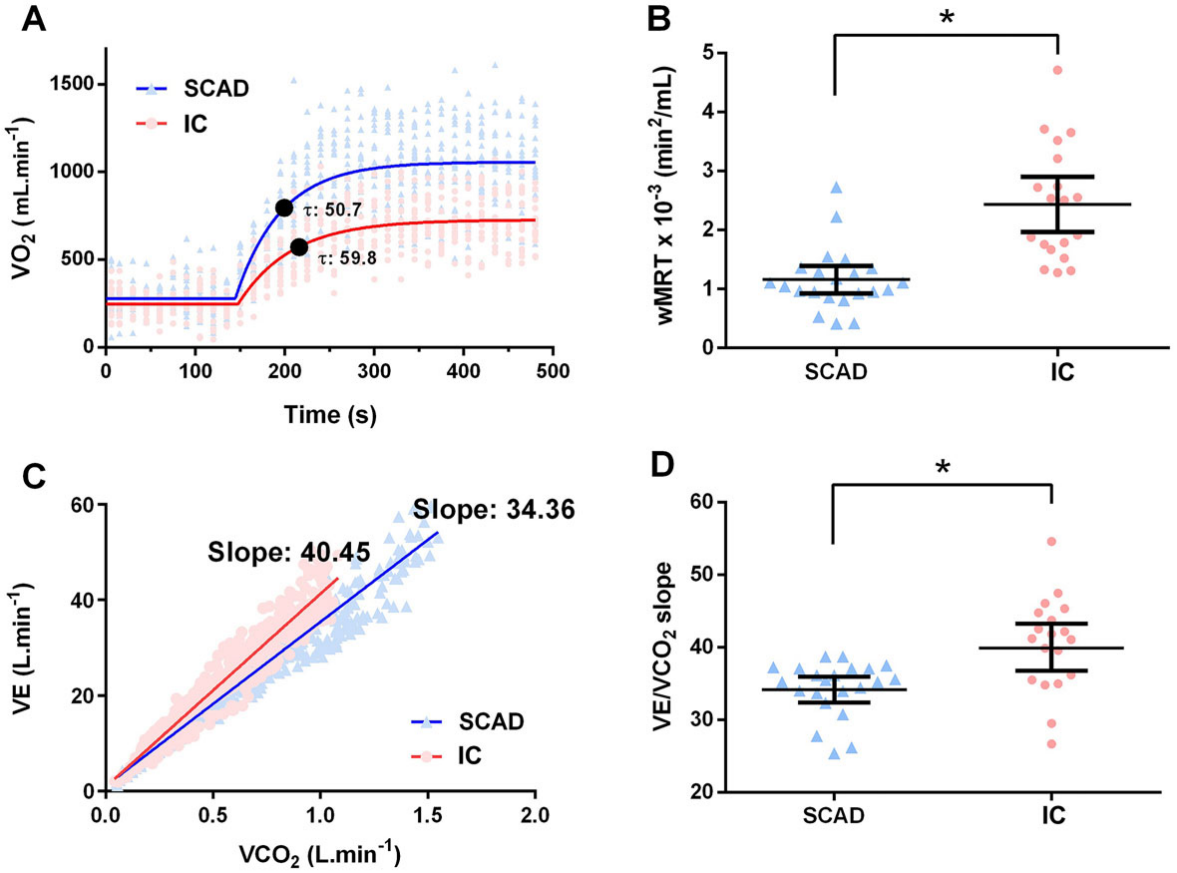
Cardiorespiratory responses according to groups. **A**, Mean response curves of oxygen uptake (VO_2_) on-kinetics and constant time “*τ*” of IC (circles) and SCAD (triangles) groups. **B**, Mean response time corrected for work rate (wMRT) was slower in IC subjects. Bars indicate mean and 95%CI. **C**, Linear regression of ventilatory efficiency (VE), i.e. slope of ventilation to carbon dioxide output ratio (VE/VCO_2_ slope) in IC (circles) and SCAD (triangles) groups. **D**, VE/VCO_2_ slope was significantly higher in patients with IC. Bars indicate mean and 95%CI. *P<0.01; Mann-Whitney U test. IC: ischemic cardiomyopathy; SCAD: stable coronary artery disease.

Mean VO_2_ on-kinetics during 6MWT was different between SCAD and IC groups (Figure 2A and B). The subjects with IC exhibited significantly lower VO_2SS_ compared to the SCAD group (710.8±30.45 [95%CI: 698.1 to 723.5] *vs* 1024±46.73 [95%CI: 1003 to 1045], P<0.0001, effect size d=7.9 and power=100%, respectively). Moreover, the IC group presented shorter wMRT compared to the SCAD group (2.43×10^-3^±0.23×10^-3^ *vs* 1.16×10^-3^±0.11×10^-3^, P<0.0001, effect size d=7.0 and power=100%), as shown in Figure 2A and B. Moreover, the VE/VCO_2_ slope was steeper in the IC group compared to the SCAD group (40.43±1.49 [95%CI: 39.76 to 41.1] *vs* 34.36±0.80 [95%CI: 34.03 to 34.69], P=0.0006, effect size d=5.1 and power=100%, respectively, Figure 2C and D).

### Relationship between LVEF and cardiorespiratory responses

**Figure 3:**
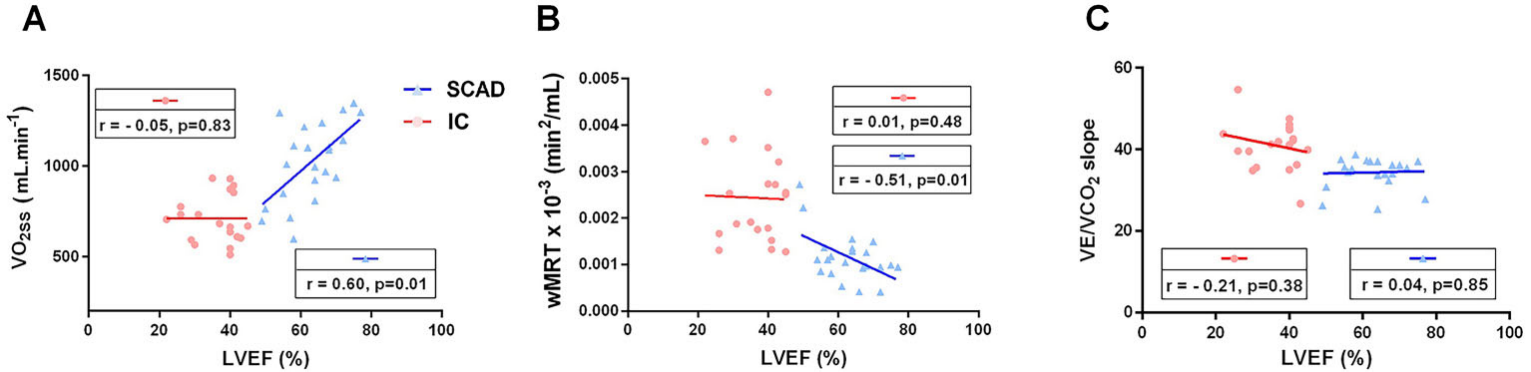
Pearson correlation of left ventricular ejection fraction (LVEF) with (**A**) steady-stable oxygen uptake (VO_2SS_), (**B**) mean response time corrected for work rate (wMRT), and (**C**) slope of ventilation to carbon dioxide output ratio (VE/VCO_2_ slope) according to SCAD (triangles) and IC (circles) groups. IC: ischemic cardiomyopathy; SCAD: stable coronary artery disease.

LVEF was moderately correlated with VO_2_ on-kinetics (VO_2SS_ and wMRT) only in the SCAD group (R^2^=0.36, P=0.01 and R^2^=0.26, P=0.01, respectively). No relationship was found between LVEF and both VO_2SS_ and wMRT on-kinetics in individuals with IC (Figure 3A and B; P=0.83 and P=0.48). No significant associations were observed between LVEF and VE/VCO_2_ slope in both groups (P>0.05).

### Forced vital capacity and ventilatory efficiency: SCAD *vs* IC

**Figure 4:**
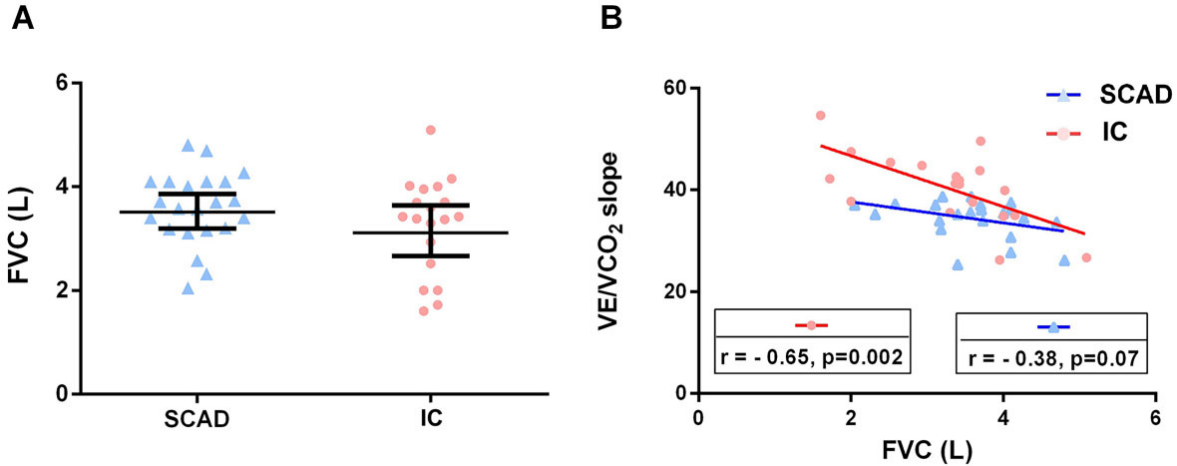
**A**, Differences in forced vital capacity (FVC) between the SCAD (triangles) and IC (circles) groups. **B**, Correlation of FVC and ventilation to carbon dioxide output ratio (VE/VCO_2_ slope) according to groups. IC: ischemic cardiomyopathy; SCAD: stable coronary artery disease. Bars indicate means and 95%CI.

FVC was similar between the groups (Figure 4A, SCAD-FVC= 3.59±0.15 *vs* IC-FVC=3.26±0.21, P=0.20). However, FVC was strongly and negatively correlated with VE/VCO_2_ slope in the IC group (Figure 4B, R ^2^=0.42, P=0.002).

## Discussion

The major finding of the present study was that during the 6MWT it was possible to detect significantly delayed VO_2_ on-kinetics in IC. Ventilatory inefficiency is more associated with pulmonary function than with LVEF. These findings suggest a potential novel application of 6MWT to more accurately evaluate submaximal exercise capacity and performance in subjects with different coronary ischemic stages.

Recent studies have evaluated cardiopulmonary and gas-exchange responses during the 6MWT in patients with chronic diseases (14,15,17). In addition to its regular application, the 6MWT is important in clinical practice due to its prognostic power and ability to differentiate the effects of therapies (18). The submaximal level of exertion of the 6MWT is better tolerated than the maximal incremental exercise testing, making it a feasible alternative to CPET for assessment of VO_2_ responses. Indeed, in the current study, the METS achieved during the 6MWT were ≅3.3 to 4.3 in both groups, and all subjects exhibited a RER below 1.15, which supports the submaximal characteristic of the test.

In the present study, the VE/VCO_2_ during the steady state was significantly higher in the IC group than in the SCAD group. This suggests that subjects with IC had a lower walking speed (as observed by lower 6MWT distance and lower METS) due to worse cardiorespiratory adaptation to an exercise.

To the best of our knowledge, this is the first study to investigate these variables during the 6MWT in patients with CAD and their possible implications in patients with moderate to severe left ventricular systolic reduction. During the 6MWT, patients are encouraged to maintain a constant walking speed achieving a steady state within 6 min, enabling the assessment of the VO_2_ on-kinetics (6). Since most activities of daily living require a repetitive transition from rest to submaximal exertion intensity, the 6MWT-VO_2_ on-kinetics provides consistent parameters. We found that *τ* and wMRT were slower and VO_2SS_ lower in patients with IC compared with the SCAD group. These findings corroborate those of a previous report where patients with a LVEF lower than 45% exhibited slower VO_2_ on-kinetics in classic constant load ergometer test (9).

Two main theories explain the factors influencing the VO_2_ on-kinetics. The ability to respond to the exercise is determined by the rate of oxygen delivery to match the demand or by the capacity of oxygen utilization by the active muscles. Considering that cardiac output is a product of stroke volume and HR, these adjustments are important to reduce the oxygen deficit by a proper response to metabolic demand during exercise (7). Moreover, patients with HF frequently exhibit a pro-inflammatory profile (19) and altered chronotropic and vasodilator reserves, which negatively impact exercise capacity (20). VO_2_ kinetics may also be altered due to systolic and/or diastolic ventricular dysfunction (8,21,22). This would be a plausible explanation to our findings, where worse responses were found in subjects with impaired ability to eject blood from the left ventricle.

In this study, these assumptions were confirmed by the low oxygen pulse (VO_2_/HR) observed in the IC group. The VO_2_/HR reflects the response of stroke volume and the amount of oxygen extracted per heartbeat during exercise (7). Although the 6MWT is a submaximal test at 30 to 40% of maximal VO_2_, the increase in VO_2_/HR is mainly affected by stroke volume. In this context, the results of our study revealed similar steady-state HR between groups. This would suggest that although achieving a lower VO_2_, the lower VO_2_/HR in the IC group could reflect impaired stroke volume increase.

Other reports suggest that HF patients have arterial-venous alterations that significantly affect exercise response (22), such as systemic vascular resistance (i.e., systemic blood flow) and/or capillary blood oxygen distribution, (i.e., amount of oxygen extraction and arterial hypoxemia) (7). The oxidative metabolic capacity of muscle fibers has been shown to be another essential contributor to VO_2_ responses (23). As CAD has an atherosclerosis etiology, the arterial-venous difference can also be impaired. In fact, a previous study reported that patients with peripheral artery disease have impaired VO_2_ kinetics (24). These assumptions indicate that both theories could explain the delayed VO_2_ on-kinetics in CAD patients observed in the present study, especially in those who already developed IC with reduced ejection fraction. Moreover, some abnormal responses observed in the SCAD group during effort suggested that dynamic monitoring of cardiopulmonary variables may help to detect hidden consequences of silent ischemia in stable coronary disease.

The VE/VCO_2_ slope, even during an ergometer constant-load exercise test, has been used previously (10). An innovative aspect of the present study was the evaluation of the VE/VCO_2_ slope during the 6MWT. The VE/VCO_2_ slope reflects the ventilatory drive by measuring the ability to increase ventilation according to CO_2_ production (25), which is currently an important prognostic marker for cardiac-related events in patients with HF (26). We found that the VE/VCO_2_ slope was different between the groups in the current study, where subjects with IC exhibited higher values.

Arena et al. (27) suggested a risk stratification model based on the VE/VCO_2_ slope_,_ known as ventilatory classification (VC) system. Our findings revealed a VE/VCO_2_ slope of 34.36 in patients with SCAD and 40.45 in those with IC. According to the VC system, it would grade our subjects, respectively, as low (VC-II, VE/VCO_2_ slope of 30.0-35.9) and moderate (VC-III, of 36.0-44.9) risk of major cardiac event. Even more representatively, our results can be interpreted by the recent data showing an expressive increased risk of mortality when the VE/VCO_2_ slope is higher than 32.8 (28).

In our study, the worse VE/VCO_2_ slope in the group with IC could be explained by altered responses, as previously described in patients with HF. The VE/VCO_2_ slope is determined by the behavior of arterial CO_2_ tension during exercise and the ratio between ventilatory dead space and tidal volume ratio (25). Therefore, the higher VE/VCO_2_ slope in patients with IC would be due to a gain in chemoreceptor response and a high ergoreceptor drive leading to an increase in VE, and also to low tidal volume due restriction during exercise as a result of left ventricular dysfunction leading to pulmonary hypertension (25). Importantly, a few studies have revealed that VE/VCO_2_ slope (26,28) and VO_2_ on-kinetics are prognostically superior to peak VO_2_ in several chronic diseases (9).

Our findings indicated that LVEF was moderately correlated with wMRT and VO_2SS_ only in the SCAD group. Surprisingly, this correlation was not observed in the IC group. This fact could suggest that, once IC is established, the LVEF value is no longer a strong determinant of exercise capacity. Similarly, a study conducted only with patients with LVEF below 50% did not find an association between LVEF and peakVO_2_ (29). This finding corroborates with a previous report, which indicates that LVEF is a weaker mortality predictor than CPET parameters (23).

Several factors influence exercise performance, such as pulmonary gas exchange capacity, pulmonary hypertension, autonomic imbalance, arterial compliance, and mainly the respiratory and peripheral muscle oxidative rate and adaptations (7). Litchfield et al. (30) reported that patients with severe IC could present compensatory mechanisms to exercise, such as tolerance to high pulmonary wedge pressure. In fact, the LVEF had no association with ventilatory efficiency in the current study. However, the FVC was associated with VE/VCO_2_ slope in IC patients. This finding suggests that pulmonary restriction at spirometry could help detect pulmonary hypertension and ventilatory inefficiency even in the absence of CPET or gas exchange analysis.

Our findings provided valuable insight into the cardiorespiratory responses during a submaximal field test, supporting the usefulness of the 6MWT and spirometry in clinical practice to assess exercise capacity and physiological alterations in subjects with IC. The present study supports the use of the 6MWT to assess VO_2_ on-kinetics and VE/VCO_2_ slope in SCAD and IC subjects. These results also reinforce the importance of properly performing the 6MWT to achieve a higher sustained walking capacity and therefore, reliably indicate submaximal exercise performance.

Despite the widespread use the 6MWT for its simplicity and cost-effectiveness, it frequently fails to identify the specific physiological causes of limited exercise capacity. The clinical relevance of our study is that it showed that the integration of wearable gas exchange sensors during the 6MWT enhanced its discriminative role, particularly in differentiating exercise limitations between patients with SCAD and IC. Our findings provided deeper insight into cardiopulmonary function, revealing delayed VO_2_ kinetics and ventilatory inefficiency in IC patients that are not captured by LVEF alone. This approach holds promise for improving patient stratification and risk assessment, ultimately optimizing patient management beyond what can be achieved with the 6MWT alone.

### Limitations

The final number of subjects included in this study was limited, despite being recruited from a tertiary-care reference institution. The restricted recruitment was due to strict exclusion criteria. Nevertheless, breath-by-breath monitoring is recognized as a sensitive and accurate method to assess VO_2_, revealing statistically significant results with statistical power above 85% with the 41 subjects.

Another limitation was related to ventilatory efficiency, since no validation of the results obtained in a 6MWT with the constant load exercise or cardiopulmonary exercise testing was made, thus this result should be interpreted with caution.

Moreover, most of the subjects were male (80%). It is well known that females have lower exercise capacity. The IC group presented a higher relative frequency of females, which may represent a bias; however, no statistical difference was observed related to gender distribution among the groups.

### Future directions

Future research should focus on including new physiological markers beyond those currently used in CAD diagnosis and clinical management, such as reduced VO_2SS_, altered cardiac function, and pulmonary dysfunction. Further research is needed to develop predictive models based on 6MWT exercise responses. These algorithms could help clinicians better forecast illness progression and personalize treatment plans to specific patients. Furthermore, additional validation of pulmonary function is required as a diagnostic tool for detecting IC patients with ventilatory inefficiency.

Large-scale cohort studies examining the evolution of exercise limitations in SCAD and IC patients over time could provide valuable insight into disease trajectory and prognosis, thus improving clinical management strategies for patients with SCAD and IC.

## Conclusions

Subjects with IC presented lower VO_2_ on-kinetics and greater ventilatory inefficiency during the 6MWT compared to those with SCAD. When LVEF falls below 45%, the severity of left ventricular dysfunction is no longer a strong determinant of exercise capacity. Pulmonary function could help identify IC patients at risk of ventilatory inefficiency and may add diagnostic power to the 6MWT.
